# Comparison of the efficacy of low, moderate and standard doses of rituximab in the treatment of membranous nephropathy

**DOI:** 10.3389/fmed.2026.1744264

**Published:** 2026-03-11

**Authors:** Mengling Guo, Wenli Wan, Jiayue Li, Shun Wu, Zhenmin Ruan, Rui Wang, Zhaoyong Zhang, Xin Geng, Xueqing Hu, Hongqi Ren

**Affiliations:** Department of Nephrology, Huaihai Hospital Affiliated of Xuzhou Medical University, Xuzhou, China

**Keywords:** efficacy, low dose, membranous nephropathy, moderate dose, rituximab, standard dose

## Abstract

**Introduction:**

Rituximab (RTX) is recommended as a first-line therapy for membranous nephropathy (MN); however, the efficacy of different dosing regimens remains controversial. This study aimed to evaluate and compare the efficacy of low-dose, moderate- and standard-dose RTX in treating MN.

**Methods:**

One hundred patients with MN who received RTX at the Affiliated Hospital of Xuzhou Medical University and Huaihai Hospital of Xuzhou Medical University between May 2021 and December 2024 were included. The patients were divided into three groups: a low-dose group (*n* = 31; 100 mg per dose), a moderate-dose group (*n* = 32; 500 mg per dose) and a standard-dose group (*n* = 37; 375 mg/m^2^ per dose). The remission rates and clinical indicators, including 24-h urinary total protein (24-UTP), serum albumin, and M-type phospholipase A2 receptor (PLA2R) levels, were compared between the three groups.

**Results:**

After 12 months of treatment, for 24 h-UTP, CD20^+^ B lymphocyte count, and anti-PLA2R-Ab: among the three groups showed significant reductions compared to baseline (*p* < 0.05). Serum albumin: among the three groups had significant increases versus baseline (*p* < 0.05); no significant changes were observed in serum creatinine or glomerular filtration rate compared to baseline (*p* > 0.05). For disease remission: the overall response rate was 67.74% in the low-dose group (*n* = 31), 65.63% in the moderate-dose group (*n* = 32) and 64.84% in the standard-dose group (*n* = 37). After 12 months, with no statistically significant difference among the three groups (*p* > 0.05). For adverse reactions: there was no significant difference in the incidence of adverse reactions among the three groups (*p* > 0.05).

**Conclusion:**

Three treatment regimens significantly reduced anti-PLA2R antibody titers and urinary protein levels, while increasing serum albumin, without a significant impact on renal function. The therapeutic efficacy of the three doses was comparable.

## 1 Introduction

Membranous nephropathy (MN) is the leading cause of nephrotic syndrome in adults over 40 years of age without diabetes, with an incidence of approximately 8–10 cases per million population (1). Idiopathic/primary membranous nephropathy (PMN), of unknown etiology, accounts for approximately 80% of cases. The pathogenesis of MN is multifactorial, with B lymphocyte-mediated humoral immune dysfunction playing a central role. Autoimmune antibodies and autoreactive B cells contribute to podocyte injury, characterized by immune complex deposition on the outer aspect of the glomerular basement membrane. This leads to marked thickening of the glomerular capillary wall and increased basement membrane permeability (2, 3), clinically manifesting as proteinuria (4). In 2009, Beck et al. (5) identified circulating autoantibodies targeting the podocyte surface phospholipase A2 receptor (PLA2R) in patients with PMN, with anti-PLA2R antibody positivity detected in 70–80% of cases. This finding established PMN as an autoimmune disease mediated by circulating autoantibodies. To date, 29 autoantigens have been identified, including thrombospondin type 1 domain-containing 7A, exostosin 1/exostosin 2, neural epidermal growth factor-like 1 protein, semaphorin 3B, and protocadherin 7 (6–8). Upon binding to podocyte antigens *in situ*, these antibodies induce podocyte injury through complement-dependent and complement-independent pathways, promoting PMN development (9).

Rituximab (RTX) is a monoclonal antibody that specifically binds to the cluster of differentiation 20 (CD20) receptor on the cell membrane of precursor and mature B cells, inducing B lymphocyte apoptosis via antibody-dependent cellular cytotoxicity and complement-dependent cytotoxicity (10). CD20 is a surface marker expressed on most B cells derived from pre-B cells before differentiation into plasmablasts. Studies have demonstrated a significant reduction in peripheral blood B cell counts following RTX therapy. The primary mechanism of RTX is the inhibition of B cell maturation, thereby preventing the formation of effector and autoreactive B cells and reducing the production of autoantibodies and other antibodies (11, 12).

According to the 2021 Kidney Disease: Improving Global Outcomes (KDIGO) guidelines, RTX is recommended as the first-line treatment for patients with MN (13). Mathew et al. (14) reported that low-dose RTX is effective in treating MN but demonstrated limited efficacy in reducing proteinuria and serum creatinine in patients with nephrotic syndrome. Similarly, Fenoglio et al. (15) found that low- and standard-dose regimens yielded favorable therapeutic outcomes. Recently, Sun et al. (16) reported that high-dose RTX may be ineffective in MN patients with significantly elevated anti-PLA2R antibody titers. Considering the variable efficacy of RTX across dosing strategies, this retrospective study was conducted to evaluate its therapeutic impact in patients with MN who were refractory to prior immunosuppressive therapy or had not received immunosuppressive treatment.

## 2 Methods

### 2.1 Study participants

This retrospective observational study included 100 patients with PMN who received RTX treatment. The patients were managed at the Department of Nephrology, The Affiliated Hospital of Xuzhou Medical University and The Affiliated Huaihai Hospital of Xuzhou Medical University, between May 2021 and July 2024.

Inclusion criteria: (1) age >18 years; (2) histopathological confirmation of PMN using renal biopsy; (3) history of prior hormone or immunosuppressant therapy or no relevant treatment before admission; (4) provision of informed consent after receiving information on RTX-related effects and risks.

Exclusion criteria: (1) MN secondary to hepatitis, diabetes, systemic lupus erythematosus, medications, malignancy, or hereditary causes; (2) presence of severe infection, cardiovascular disease, hepatic dysfunction, or hematological disorders; (3) immunodeficiency or immunocompromised state (e.g., human immunodeficiency virus infection); (4) history of hypersensitivity to similar agents or known allergic constitution; (5) mental health conditions impairing treatment compliance.

Written informed consent was obtained from all participants. The study was approved by the Ethics Committee of Huaihai Hospital Affiliated to Xuzhou Medical University (LL-2024YX01).

### 2.2 Study design

Patients were allocated into three groups based on RTX dosing regimens: the low-dose group was administered as follows: 100 mg per week for a single dose, totaling 4 doses, with a cumulative dose ≤1,200 mg, the moderate-dose group received 500 mg per infusion once weekly for 4 consecutive weeks (17, 18), while the standard-dose group received 375 mg/m^2^ via intravenous infusion once weekly for 4 weeks (13, 19). All patients received pretreatment with 100 mg of methylprednisolone and 25 mg of promethazine hydrochloride before each RTX infusion.

### 2.3 Observation data and efficacy evaluation

General characteristics [age, sex, body mass index (BMI), and history of hypertension] and clinical parameters [including triglycerides, total cholesterol, glomerular filtration rate, uric acid, serum urea, anti-PLA2R antibody, serum albumin, serum creatinine, 24-h urinary total protein (24-UTP), and hemoglobin levels] were compared among the three groups at baseline and at 1, 3, 6, and 12 months post-treatment. Serum PLA2R antibody levels were measured using enzyme-linked immunosorbent assay. All patients in both groups received continuous RTX treatment for at least 12 months. Clinical remission rates were evaluated at 12 months following RTX infusion.

Efficacy evaluation 12 months after initial RTX infusion:

Treatment remission was categorized as complete remission (CR) or partial remission (PR), defined as follows:

CR: 24-UTP <300 mg or urinary albumin/creatinine ratio <300 mg/g, with stable renal function and serum albumin >35 g/L.
PR: >50% reduction in 24-UTP from baseline, with 24-UTP reduced to 0.3–3.5 g or urinary protein/creatinine ratio of 300–3,500 mg/g.

Clinical remission was defined as the combined total of CR and PR. Non-response was defined as failure to meet CR or PR criteria. The overall response rate was calculated as [(CR + PR)/total cases] × 100% (3).

### 2.4 Statistical analysis

Statistical analyses were performed using SPSS 25.0 software. Normally distributed continuous variables are expressed as mean ± standard deviation, with intergroup comparisons performed using the independent samples t-test and intragroup comparisons using the paired samples t-test. Skewed continuous data are presented as median (interquartile range), and intergroup comparisons were performed using the Mann–Whitney *U* test. Kaplan–Meier curves combined with the log-rank test to analyze the temporal differences between PLA2R serological remission and clinical remission. Categorical variables are expressed as count (%) and analyzed using the chi-square test. A two-sided *p*-value of <0.05 was considered statistically significant.

## 3 Results

### 3.1 Baseline data comparison

One hundred eligible patients were enrolled, including those previously treated with hormones (*n* = 4) or immunosuppressants [cyclophosphamide (*n* = 5), cyclosporine (*n* = 1), and tacrolimus (*n* = 16)], as well as untreated patients (*n* = 74). The mean age was 48.04 ± 12.53 years. No statistically significant differences were observed among the three groups in baseline variables, including age, sex, BMI, hypertension, 24-UTP, anti-PLA2R antibody levels, CD20^+^ B lymphocyte count, serum albumin, blood urea nitrogen, serum creatinine, serum uric acid, estimated glomerular filtration rate (eGFR), triglycerides, total cholesterol, white blood cells, red blood cells, hemoglobin, platelets, immunoglobulin (Ig) A, IgG, and IgM (*p* > 0.05), indicating baseline comparability (Table 1).

**Table 1:** Baseline data comparison among the three dosage groups.

Variables	Total (*n* = 100)	Low dose group (*n* = 31)	Moderate dose group (*n* = 32)	Standard dose group (*n* = 37)	*p*
Age (years), mean ± SD	48.04 ± 12.53	47.58 ± 13.71	46.28 ± 10.59	49.11 ± 12.96	0.644
Sex, male (%)	66/34	20/11	23/9	23/14	0.685
BMI (kg/m^2^), mean ± SD	25.45 ± 3.59	26.00 ± 3.71	26.34 ± 3.49	24.35 ± 3.38	0.053
24 h-UTP (g/24 h), medium (IQR)	3.66 (1.88, 6.40)	2.51 (1.00, 5.73)	3.41 (1.82, 6.74)	4.41 (3.26, 6.32)	0.081
WBC (×10^9^/L), mean ± SD	6.99 ± 2.22	7.73 ± 2.23	6.55 ± 2.07	6.83 ± 2.25	0.110
N (×10^9^/L), medium (IQR)	3.90 (3.07, 5.98)	4.87 (3.52, 6.41)	3.70 (2.73, 5.60)	3.67 (2.98, 5.73)	0.097
RBC (×10^12^/L), mean ± SD	4.40 ± 0.64	4.31 ± 0.64	4.50 ± 0.67	4.38 ± 0.59	0.539
Hb (g/L), mean ± SD	133.30 ± 18.96	129.44 ± 21.60	138.25 ± 17.80	131.71 ± 17.31	0.171
PLT (×10^9^/L), mean ± SD	244.00 ± 68.67	251.19 ± 59.87	245.75 ± 76.74	236.65 ± 68.49	0.707
L (×10^9^/L), mean ± SD	1.90 ± 0.69	1.89 ± 0.73	1.93 ± 0.71	1.88 ± 0.66	0.950
TP (g/L), mean ± SD	52.01 ± 8.79	52.31 ± 10.00	52.43 ± 7.72	51.40 ± 8.91	0.874
ALB (g/L), mean ± SD	29.29 ± 7.31	30.66 ± 8.73	29.20 ± 6.26	28.28 ± 7.00	0.442
eGFR (mL/min), medium (IQR)	109.52 (95.69, 116.14)	105.75 (62.45, 114.85)	111.05 (106.56, 120.00)	109.05 (91.72, 117.94)	0.082
SCr (μmol/L), medium (IQR)	63.00 (52.25, 75.75)	66.50 (49.50, 111.75)	59.50 (50.50, 68.00)	65.00 (56.00, 74.75)	0.076
UA (μmol/L), mean ± SD	352.81 ± 97.69	342.54 ± 90.75	366.20 ± 103.71	348.89 ± 98.79	0.620
BUN (mmol/L), medium (IQR)	5.43 (4.27, 6.54)	5.72 (5.02, 8.52)	5.41 (4.30, 6.22)	5.32 (3.97, 6.45)	0.168
ALT (U/L), medium (IQR)	17.00 (13.75, 22.25)	17.00 (13.00, 20.00)	17.00 (14.00, 22.00)	19.00 (13.25, 24.75)	0.574
AST (U/L), mean ± SD	20.09 ± 7.67	18.15 ± 3.86	18.80 ± 5.28	22.56 ± 10.51	0.052
TG (mmol/L), medium (IQR)	1.83 (1.32, 2.70)	1.81 (1.26, 2.45)	1.95 (1.26, 3.47)	1.83 (1.58, 3.20)	0.455
TC (mmol/L), mean ± SD	6.99 ± 2.57	5.82 ± 1.56	7.61 ± 3.32	7.49 ± 2.17	0.075
Anti-PLA2R Ab (RU/mL), medium (IQR)	44.00 (1.00, 183.00)	18.00 (3.21, 195.50)	34.00 (1.00, 138.00)	54.00 (6.00, 194.50)	0.749
CD20^+^ B cell (cells/μL), medium (IQR)	198.00 (88.00, 316.00)	93.00 (23.75, 254.75)	213.00 (144.50, 511.50)	217.00 (105.00, 323.00)	0.083
IgA (g/L), mean ± SD	1.96 ± 0.92	1.54 ± 0.58	2.40 ± 0.96	1.91 ± 0.96	0.087
IgG (g/L), mean ± SD	6.38 ± 2.51	6.13 ± 2.07	6.30 ± 2.84	6.60 ± 2.59	0.815
IgM (g/L), mean ± SD	0.81 (0.60, 1.15)	0.60 (0.52, 0.95)	0.99 (0.62, 1.69)	0.83 (0.70, 1.06)	0.053
C3 (g/L), mean ± SD	1.16 ± 0.16	1.08 ± 0.13	1.15 ± 0.16	1.19 ± 0.16	0.238
C4 (g/L), mean ± SD	0.29 ± 0.09	0.27 ± 0.08	0.28 ± 0.09	0.30 ± 0.10	0.626

BMI, body mass index; 24-UTP, 24-h urinary protein quantification; WBC, white blood cell; N, neutrophil; RBC, red blood cell; Hb, hemoglobin; PLT, platelet; L, lymphocyte; TP, total serum protein; ALB, serum albumin; eGFR, estimated glomerular filtration rate; SCr, serum creatinine; UA, uric acid; BUN, blood urea nitrogen; AST, aspartate aminotransferase; ALT, alanine aminotransferase; TG, triglycerides; TC, total cholesterol; Anti-PLA2R Ab, anti-phospholipase A2 receptor antibody; CD20^+^ B cell, CD20^+^ B lymphocyte count; IgA, immunoglobulin A; IgG, immunoglobulin G; IgM, immunoglobulin M; C3, complement 3; C4, complement 4.

### 3.2 Clinical and immunological outcomes

**Figure 1:**
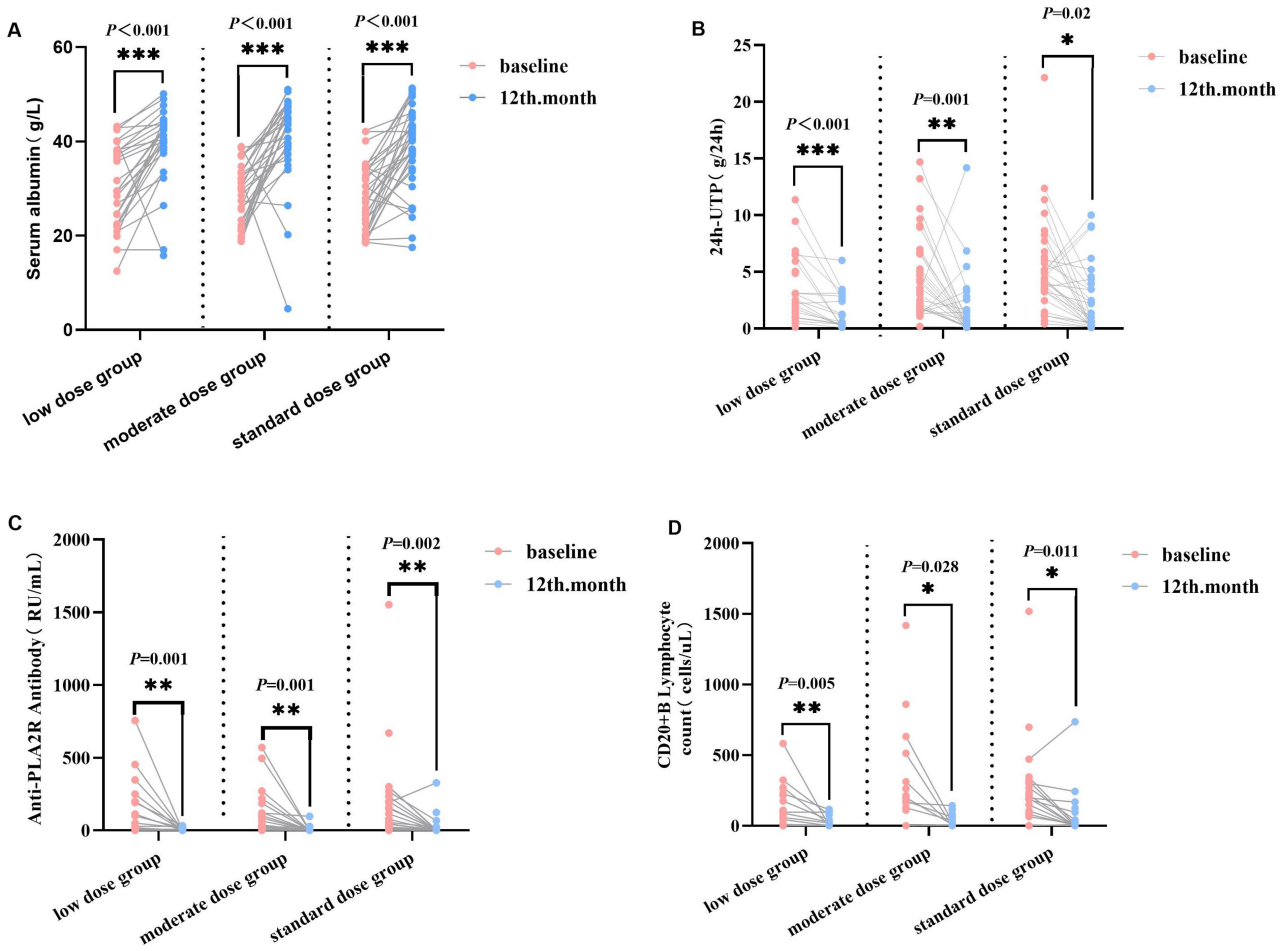
Clinical and immunological outcomes after 12-month RTX therapy among three groups. This figure illustrates the changes in **(A)** serum albumin, **(B)** 24 h-UTP, **(C)** anti-PLA2R antibody titers, and **(D)** CD20^+^ B lymphocyte counts at baseline and after 12 months of treatment with low-dose, moderate-dose, or standard-dose RTX. Each line represents the individual data trajectory of one patient, and the horizontal bars represent the median values for each group. Intra-group comparisons: paired *t*-test or Wilcoxon signed-rank test was used to analyze differences between baseline and 12-month values within the same treatment group. ^*^p < 0.05, ^**^p < 0.01, and ^***^p < 0.001 vs. baseline. RTX, rituximab; 24 h-UTP, 24-h urinary total protein; anti-PLA2R, anti-phospholipase A2 receptor antibody.

Serum albumin: No significant differences in serum albumin were observed among the three groups before treatment and at 12 months after RTX treatment (*p* > 0.05). Intra-group comparisons showed that serum albumin increased from 30.66 ± 8.73 g/L to 39.35 ± 7.84 g/L in the low-dose group (*p* < 0.0001), from 29.20 ± 6.26 g/L to 40.73 ± 9.70 g/L in the moderate-dose group (*p* < 0.001), and from 28.28 ± 7.00 g/L to 38.11 ± 8.60 g/L in the standard-dose group (*p* < 0.001) (Figure 1).
24-UTP: No significant differences in 24 h-UTP were observed among the three groups before treatment (*p* > 0.05). After 12 months of RTX treatment, significant intra-group improvements were detected (*p* < 0.05): In the low-dose group, 24 h-UTP decreased from 2.51 (1.00, 5.73) g/24 h to 0.47 (0.26, 2.68) g/24 h (*p* < 0.001); in the moderate-dose group, from 3.41 (1.82, 6.74) g/24 h to 0.70 (0.14, 2.10) g/24 h (*p* = 0.01); and in the standard-dose group, from 4.41 (3.26, 6.32) g/24 h to 1.17 (0.25, 4.26) g/24 h (*p* < 0.05) (Figure 1).
CD20^+^ B cell count: No significant differences in CD20^+^ B-cell count were observed among the three groups before treatment and at 12 months after rituximab (RTX) treatment (*p* > 0.05). Intra-group comparisons revealed that CD20^+^ B-cell count decreased from 93.00 (23.75, 254.75) cells/μL to 23.50 (0.00, 99.00) cells/μL in the low-dose group (*p* < 0.01), from 213.00 (144.50, 511.50) cells/μL to 33.50 (3.00, 70.25) cells/μL in the moderate-dose group (*p* < 0.05), and from 217.00 (105.00, 323.00) cells/μL to 13.00 (0.00, 41.00) cells/μL in the standard-dose group (*p* < 0.05) (Figure 1).
Anti-PLA2R-Ab: No significant differences in anti-PLA2R-Ab levels were observed among the three groups before treatment and at 12 months after rituximab (RTX) treatment (all *p* > 0.05). Intra-group comparisons showed that anti-PLA2R-Ab decreased from 18.00 (3.71, 195.50) RU/mL to 1.00 (1.00, 4.25) RU/mL in the low-dose group (*p* < 0.01), from 34.00 (2.00, 138.00) RU/mL to 1.00 (1.00, 1.00) RU/mL in the medium-dose group (*p* < 0.01), and from 54.00 (6.50, 194.50) RU/mL to 1.00 (1.00, 1.00) RU/mL in the standard-dose group (*p* < 0.01) (Figure 1).
Renal function: 12 months post-treatment, there were no significant changes in eGFR and serum creatinine levels relative to baseline across all three groups (*p* > 0.05).

### 3.3 Adverse events

Twenty patients experienced adverse events during or after RTX infusion. These included 10 cases of infection (four in the low-dose group, three in the moderate-dose group and three in the standard-dose group), manifesting as pulmonary infection (cough and expectoration) or urinary tract infection (urinary frequency and urgency). Three cases had abnormal liver function (two in the low-dose group and one patient in the moderate-dose group) all manifested as mildly elevated transaminase levels. Gastrointestinal reactions occurred in two patients (one in the moderate-dose group and one in the standard group), both presenting with diarrhea. Additional events in the low-dose group included one case of chills, which occurred during the infusion and one case of herpes, presenting as herpes simplex (which manifested as mild perioral herpes one day after RTX infusion, without itching or pain. Symptoms were significantly relieved after topical ointment application). Additional events in the standard-dose group included one case each of dizziness, skin rash, and bilateral lower limb edema. No statistically significant difference in the incidence of adverse events was observed among the three groups (*p* > 0.05) (Table 2).

**Table 2:** Comparison of adverse events across groups.

Adverse events	Low dose group (*n* = 31)	Moderate dose group (*n* = 32)	Standard dose group (*n* = 37)	Total (*n* = 100)	*χ* ^2^	*p*
Infection	4 (12.9)	3 (9.38)	3 (8. 11)	10 (10)		
Liver dysfunction	2 (6.45)	1 (3. 13)	0 (0)	3 (3)		
Gastrointestinal reactions	0 (0)	1 (3. 13)	1 (2.70)	2 (2)		
Dizziness	0 (0)	0 (0)	1 (2.70)	1 (1.45)		
Chill	1 (3.23)	0 (0)	0 (0)	1 (1.45)	1.063	0.588
Herpes	0 (0)	0 (0)	1 (2.70)	1 (1.45)		
Skin rash	1 (3.23)	0 (0)	0 (0)	1 (1)		
Edema	0 (0)	0 (0)	1 (2.70)	1 (1)		
Total (%)	8	5 (15.63)	7 (18.92)	20 (20)		

Infection manifesting as pulmonary infection (cough and expectoration) and urinary tract infection (frequency and urgency of urination). Liver dysfunction, characterized by elevated transaminases. Gastrointestinal reactions, presenting as diarrhea. Chills occurred during the infusion. Herpes presenting as herpes simplex. Skin rash presents as erythema on the extremities with mild pruritus. Edema manifesting as bilateral lower limb edema.

### 3.4 Efficacy assessment

#### 3.4.1 Serological remission

**Figure 2:**
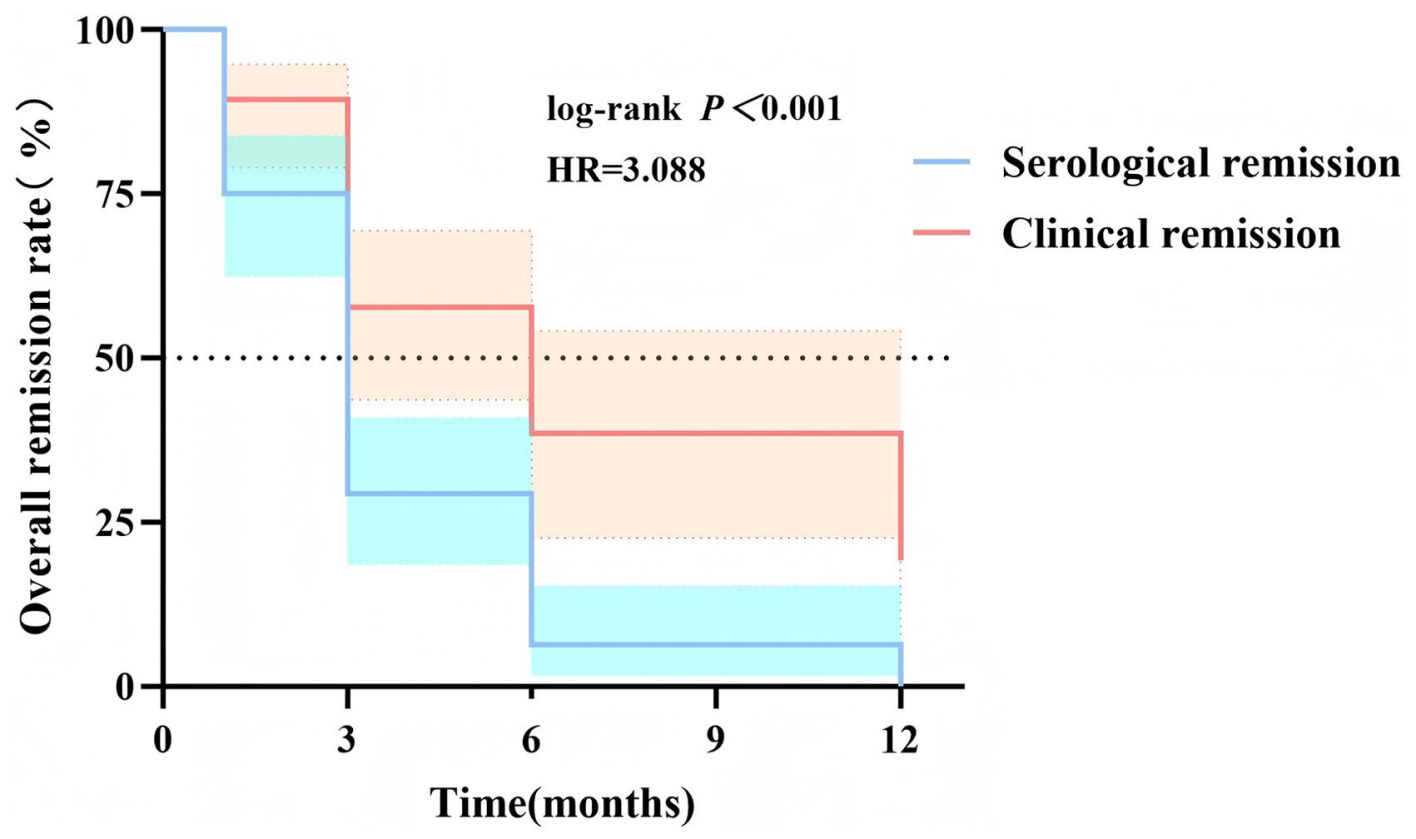
Kaplan–Meier analysis of overall remission rates in patients with MN during 12-month follow-up: comparison between serological remission and clinical remission. This figure shows the cumulative overall remission rates for patients with MN over a 12-month follow-up period. The blue curve represents the cumulative rate of serological remission, while the red curve represents the cumulative rate of clinical remission. The log-rank test was used to compare the differences between the two remission groups, revealing a statistically significant difference [log-rank *p* < 0.001; HR = 3.088, 95% CI (1.786–5.342)]. MN, membranous nephropathy; HR, hazard ratio; CI, confidence interval.

All patients were followed up for more than 12 months. At the 12-month follow-up, among patients with positive PLA2R antibody, 18 patients in the low-dose group, 23 patients in the moderate-dose group, and 25 patients in the standard-dose group achieved serological remission, with no significant difference among groups (*χ*^2^ = 0.806, *p* = 0.668). Kaplan–Meier survival analysis showed that the median time to PLA2R serological remission was 3 months, which was earlier than the median time to clinical remission (6 months), and the difference was statistically significant (log-rank *χ*^2^ = 16.27, *p* < 0.0001). The hazard ratio (HR) was 3.088 (95% CI: 1.786–5.342) (Figure 2).

#### 3.4.2 Clinical remission

All patients were followed up over 12 months. By the end of the follow-up, 21 patients in the low-dose group, 21 patients in the moderate-dose group and 24 patients in the standard-dose group had achieved CR or PR. No statistically significant difference in the overall remission rates was observed among the three groups (*χ*^2^ = 0.968, *p* = 0.065) (Table 3).

**Table 3:** Comparison of efficacy outcomes at 12-month follow-up.

	Overall effective at 1 month	Overall effective at 3 month	Overall effective at 6 month	Overall effective at 12 month
Low dose group (*n* = 31)	5 (16.13)	12 (38.71)	20 (64.52)	21 (67.74)
Moderate dose group (*n* = 32)	12 (37.50)	10 (31.25)	20 (62.50)	21 (65.63)
Standard dose group (*n* = 37)	4 (10.81)	12 (32.43)	23 (62.16)	24 (64.86)
*χ* ^2^	8.010	0.455	0.045	0.065
*p*	0.018	0.797	1.000	0.968

CR, complete remission; PR, partial remission; ineffective: failure to meet CR or PR criteria; overall response rate: [(number of CR + number of PR)/total cases] × 100%.

## 4 Discussion

In recent years, the incidence of MN has been increasing annually. Approximately one-third of patients achieve spontaneous remission, one-third progress to end-stage renal disease (ESRD), and the remainder develop non-progressive chronic kidney disease (20). The core pathogenesis of MN mainly involves the *in situ* binding of autoantibodies against podocyte antigens (such as anti-PLA2R) to form subepithelial immune complexes (ICs), which then deposit and become embedded in the glomerular basement membrane (GBM), leading to persistent podocyte injury. Podocyte injury represents the direct cellular basis underlying proteinuria in MN. Studies have confirmed that with appropriate disease management, fewer than 10% of patients progress to ESRD within 10 years (21).

The treatment strategy for MN consists primarily of supportive care and immunosuppressive therapy. Classic immunosuppressive agents include glucocorticoids, cyclophosphamide, tacrolimus, and cyclosporine A. Although immunosuppressive therapy effectively controls disease activity, it is associated with high costs, an increased risk of infection and renal function impairment, a high relapse rate, and reduced quality of life in patients (22). Therefore, optimizing therapeutic regimens remains a major focus of current research.

RTX acts against MN mainly through the following mechanisms: (1) It specifically depletes CD20^+^ B cells, inhibits plasma cell activation and the production of pathogenic autoantibodies, thereby reducing the formation of new immune complexes and promoting the degradation and resorption of deposited immune complexes, ultimately interrupting podocyte injury. (2) By reducing immune complex deposition and complement activation, RTX alleviates local inflammatory stress in renal tissue, upregulates the protective Sirt6 pathway, and inhibits the overactivation of the Wnt1/*β*-catenin and RAS pathways. At the signaling pathway level, this stabilizes podocyte structure and function, reduces proteinuria, and delays renal function deterioration (23). (3) It suppresses abnormally expanded B-cell clones in overlapping nephropathies, reduces excessive production of immunoglobulins such as IgG and IgA, alleviates immune complex deposition in the subepithelial and mesangial areas, and improves the renal immune microenvironment (2, 24, 25).

According to the KDIGO 2021 guidelines, RTX is recommended as a first-line therapeutic agent for patients with MN (13). However, consensus has not yet been reached regarding its optimal dosage.

This study first compared the baseline values and follow-up data of patients treated with different doses of RTX. We found that RTX is one of the effective agents for the treatment of MN, and that different doses of RTX can significantly reduce anti-PLA2R antibody titers and urinary protein levels, as well as increase serum albumin levels. However, there were no significant differences in therapeutic efficacy among the three dosage regimens (*p* > 0.05). In the study by Fenoglio et al. (15), membranous nephropathy was treated with regimen 1 (one dose of RTX 375 mg/m^2^), regimen 2 (four weekly doses of RTX 375 mg/m^2^), and regimen 3 (Ponticelli’s regimen). At 24 months of follow-up, significant improvements in urinary protein levels were observed in patients receiving all three regimens, with no significant difference in remission rates among the three groups. This indicates that RTX is a promising alternative to the Ponticelli regimen. Even the low-dose RTX is equally applicable, which is largely consistent with the conclusions of the present study. Recently, Xu et al. (26) proposed an individualized rituximab regimen: low-dose rituximab (guided by peripheral B cell counts and anti-PLA2R antibody levels) versus the standard treatment regimen (375 mg/m^2^ weekly for 4 weeks or 1,000 mg on days 0 and 15) for the treatment of refractory membranous nephropathy (RMN). The individualized regimen (low-dose regimen) is a cost-effective and safe alternative therapy for patients with PMN. However, a higher proportion of patients enrolled in this study were refractory PMN cases with a history of prior immunosuppressive therapy, which constitutes a non-negligible confounding factor.

Relevant studies have shown (18, 27) that in the treatment of membranous nephropathy MN, low-dose RTX regimens (100 mg weekly for 4 consecutive weeks, 200 mg weekly for 4 consecutive weeks, or a total RTX dose of <1,200 mg within 6 months) exert significant efficacy in promoting disease remission with a single low-dose administration. No significant difference was observed in therapeutic efficacy between these low-dose regimens and standard-dose regimens (375 mg/m^2^ intravenously once weekly for 4 weeks, or 1 g intravenously on days 0 and 15). The conclusions of the present study support this view.

In addition, several studies have demonstrated that RTX achieves a high clinical remission rate in patients with MN, including those with renal insufficiency or advanced age, with relatively mild effects on renal function and maintenance of stable renal function. These findings are consistent with the results of the present study (28–31), suggesting that RTX therapy may also be an alternative treatment for reducing proteinuria and maintaining stable renal function.

A recent case report indicated that Kimura’s disease can be accompanied by both MN and IgA nephropathy, with a common pathological basis involving polyclonal B-cell hyperactivation, increased autoantibodies and immune complexes, among others. The B-cell-targeting effect of RTX is not only applicable to classic idiopathic membranous nephropathy, but also suppresses abnormally expanded B-cell clones in such overlapping nephropathies, reduces excessive production of immunoglobulins such as IgG and IgA, alleviates immune complex deposition in the subepithelial and mesangial areas, and improves the renal immune microenvironment. This mechanistically supports RTX as an effective therapeutic approach for refractory membranous nephropathy with multiple comorbidities and complex immune disorders (32).

Regarding adverse reactions, most patients tolerated RTX well. Adverse reactions occurred in 20% of patients, with no significant difference in the incidence rate among the three groups (*χ*^2^ = 1.063; *p* = 0.588), which is similar to the 22.2% incidence rate reported in previous studies (31). Meanwhile, only one patient experienced an infusion reaction (chills during infusion), which differs from previous studies indicating that infusion reactions are the most common adverse reaction (33) Possible reasons for this result are as follows: (1) All patients received premedication with methylprednisolone and promethazine hydrochloride before infusion to inhibit immune responses, alleviate allergic and inflammatory reactions, thereby reducing the incidence and severity of infusion reactions. (2) Slow infusion rate: a slow infusion allows the body more time to adapt to the drug, reducing the release of cytokines and other chemical mediators, which in turn lowers the risk of infusion reactions. One case presented with herpes infection, characterized by perioral herpes that occurred 1 day after RTX infusion, with no pain or pruritus. The symptoms completely resolved after topical application of antiviral ointment. Regarding serious adverse events, relevant data in the present study were carefully verified, and no malignant or fatal events were identified. The findings of Hao et al. (18) showed that the incidence of adverse reactions in the single low-dose group (200 mg per dose, once weekly) was 13.33%, which was lower than that in the conventional-dose group (500 mg per dose, once weekly) at 50%. This further supports the safety profile of low-dose RTX.

The production of anti-PLA2R antibodies is one of the key initiating factors in the pathogenesis of MN (34). A study by Seitz-Polski et al. (35) demonstrated that anti-PLA2R antibodies can serve as an early efficacy marker for PMN, with changes preceding the remission of proteinuria. If the antibody becomes positive or its titer increases, disease recurrence may occur. Additionally, multiple studies (20, 36, 37) have confirmed that anti-PLA2R antibodies can be used as an indicator to monitor the efficacy of RTX. Changes in anti-PLA2R antibody titer (either increase or decrease) help predict the therapeutic efficacy and prognosis of RTX treatment. Therefore, anti-PLA2R antibodies play a crucial role in the progression and prognosis of MN. In the present study, the serum anti-PLA2R antibody level in MN patients was significantly reduced compared with the baseline level after RTX treatment. Additionally, a positive correlation was observed between anti-PLA2R antibody titer and quantitative proteinuria (Spearman’s *ρ* = 0.465, *p* < 0.01). This indicates that the higher the antibody titer, the more severe the proteinuria tends to be; conversely, the lower the antibody titer, the milder the proteinuria, and it may even gradually remit to the normal range, thereby supporting the aforementioned view. However, due to the short follow-up duration and insufficient monitoring of anti-PLA2R antibody, our understanding of the association between dynamic changes in the antibody and long-term prognosis may be limited.

We defined serological remission as a marked reduction in PLA2R antibody titer (≥50%) from baseline or undetectable levels (<2 RU/mL) (38). Using Kaplan–Meier survival curve analysis, we found that the median time to serological remission in this study was 3 months, which was significantly earlier than the median time to clinical remission (6 months). These results were highly consistent with those reported in several previous studies (39, 40), confirming that serological remission can serve as an important early predictor of clinical remission in the treatment of membranous nephropathy. Our findings also validate the KDIGO guideline that patients who achieve serological remission within 3 months of treatment have a significantly higher probability of attaining clinical remission within the subsequent 6–12 months (13).

This study was a retrospective, single-center investigation. During follow-up, some patients dropped out due to disease progression, poor compliance, and other reasons. In addition, inaccurate information resulted from vague recall of past medical history and medication use. These factors may have introduced bias into the clinical data. The present study has several limitations. The sample size was relatively small, and the follow-up duration was 12 months, so long-term outcomes such as disease relapse and renal function deterioration were not observed, making it impossible to evaluate the long-term efficacy and safety of RTX. The uneven distribution of some baseline indicators may have potentially affected the assessment of therapeutic efficacy, leading to certain confounding factors in the intergroup comparison of treatment effects.

In the future, multicenter collaboration is needed to further expand the sample size and extend the follow-up period. Optimizing randomization or adopting methods such as propensity score matching to balance baseline characteristics will help verify the longer-term efficacy of different doses of RTX. We also suggest that future studies should focus on the long-term efficacy of low-dose RTX in the treatment of MN, especially the differences in efficacy among patients with different immune statuses and renal function levels. Furthermore, exploring the relationship between changes in serum anti-PLA2R antibody titers and treatment response may provide more definite guidance for individualized therapy. In conclusion, our study demonstrates that low-dose, moderate-dose, and standard-dose RTX all exhibit similar therapeutic efficacy in the treatment of MN. This provides a new perspective for the clinical selection of RTX dosage, with low-dose RTX offering potential cost-effectiveness. Selecting an appropriate RTX dosage based on patients’ disease severity, immune status, and economic conditions may be crucial in clinical practice. With the advancement of further research, individualized RTX treatment regimens are expected to bring better prognosis to patients.

## Data Availability

The raw data supporting the conclusions of this article will be made available by the authors, without undue reservation.
